# Intensity of end-of-life care among children with life-threatening conditions: a national population-based observational study

**DOI:** 10.1186/s12887-023-04186-9

**Published:** 2023-07-24

**Authors:** Kimberley Widger, Sarah Brennenstuhl, Katherine E. Nelson, Hsien Seow, Adam Rapoport, Harold Siden, Christina Vadeboncoeur, Sumit Gupta, Peter Tanuseputro

**Affiliations:** 1 Lawrence S. Bloomberg Faculty of Nursing, University of Toronto, Toronto, Canada; 2 Hospital for Sick Children, Toronto, Canada; 3 McMaster University, Hamilton, Canada; 4 Emily’s House Children’s Hospice, Toronto, Canada; 5 Canuck Place Children’s Hospice, Vancouver, Canada; 6 British Columbia Children’s Hospital, Vancouver, Canada; 7 Faculty of Medicine, University of British Columbia, Vancouver, Canada; 8 Faculty of Medicine, University of Ottawa, Ottawa, Canada; 9 CHEO, Ottawa, Canada; 10 Roger Neilson House, Ottawa, Canada; 11 Ottawa Hospital Research Institute, Ottawa, Canada

**Keywords:** End-of-life care, High intensity care, Life-threatening conditions, Pediatrics

## Abstract

**Background:**

Children with life-threatening conditions frequently experience high intensity care at the end of life, though most of this research only focused on children with cancer. Some research suggests inequities in care provided based on age, disease type, socioeconomic status, and distance that the child lives from a tertiary hospital. We examined: 1) the prevalence of indicators of high intensity end-of-life care (e.g., hospital stays, intensive care unit [ICU] stays, death in ICU, use of cardiopulmonary resuscitation [CPR], use of mechanical ventilation) and 2) the association between demographic and diagnostic factors and each indicator for children with any life-threatening condition in Canada.

**Methods:**

We conducted a population-based retrospective cohort study using linked health administrative data to examine care provided in the last 14, 30, and 90 days of life to children who died between 3 months and 19 years of age from January 1, 2008 to December 31, 2014 from any underlying life-threatening medical condition. Logistic regression was used to model the association between demographic and diagnostic variables and each indicator of high intensity end-of-life care except number of hospital days where negative binomial regression was used.

**Results:**

Across 2435 child decedents, the most common diagnoses included neurology (51.1%), oncology (38.0%), and congenital illness (35.9%), with 50.9% of children having diagnoses in three or more categories. In the last 30 days of life, 42.5% (*n* = 1035) of the children had an ICU stay and 36.1% (*n* = 880) died in ICU. Children with cancer had lower odds of an ICU stay (OR = 0.47; 95% CI = 0.36–0.62) and ICU death (OR = 0.37; 95%CI = 0.28–0.50) than children with any other diagnoses. Children with 3 or more diagnoses (vs. 1 diagnosis) had higher odds of > 1 hospital stay in the last 30 days of life (OR = 2.08; 95%CI = 1.29–3.35). Living > 400 km (vs < 50 km) from a tertiary pediatric hospital was associated with higher odds of multiple hospitalizations (OR = 2.09; 95%CI = 1.33–3.33).

**Conclusion:**

High intensity end of life care is prevalent in children who die from life threatening conditions, particularly those with a non-cancer diagnosis. Further research is needed to understand and identify opportunities to enhance care across disease groups.

**Supplementary Information:**

The online version contains supplementary material available at 10.1186/s12887-023-04186-9.

## Background

Frequent use of high intensity interventions such as admission to the intensive care unit (ICU), hospital stays, use of mechanical intervention, and death in hospital have been demonstrated over the last 30 days of life in children with cancer across several studies and countries [1–9]. Use of high intensity interventions may increase symptom burden and reduce quality of life [10–12] which have been associated with complicated grief and depression in parents following the child’s death [13]. Potential disparities have been noted in the use of these interventions including higher use in younger children [7, 9], in those with leukemia versus other cancer types [1, 3, 6, 7], in children from minority groups [7], or who were socially [3] or economically disadvantaged [2], and in children who lived closer to the treating hospital [7].

While cancer remains a leading cause of death, it accounts for only a quarter of life-threatening conditions in children [14]. Other conditions include those similar to cancer where curative treatments may fail (e.g., organ failure); those where life-prolonging treatments are available but are ultimately fatal (e.g., Duchenne muscular dystrophy); progressive conditions (e.g., severe metabolic conditions); and static but irreversible conditions where complications may result in death (e.g., severe cerebral palsy) [15]. While a few studies have been conducted in children with these wider range of conditions to examine the use of high intensity end-of-life (EOL) interventions and the potential influence of sociodemographic factors [16–18], the question has not been evaluated with national population-based data.

Analysis of health administrative data may reveal patient characteristics associated with needs, optimal/sub-optimal care, and opportunities to optimize service use and enhance care quality across the full population of children with life-threatening conditions. We examined the prevalence of indicators of high intensity EOL care in the last 14, 30, and 90 days of life and the association between demographic and diagnostic factors and each indicator of high intensity care for children who died from a life-threatening condition in Canada.

## Methods

### Population and cohort

In this observational, retrospective cohort study we examined the population of Canadian children who died between 3 months and 19 years of age from January 1, 2008 through December 31, 2014 from an underlying life-threatening medical condition using linked health administrative datasets: Canadian Vital Statistics Database (CVSD), Discharge Abstracts Database (DAD) and National Ambulatory Care Reporting System (NACRS). The CVSD is a census of all deaths occurring in Canada each year, with relevant demographic and cause of death information coded using the International Classification of Diseases, 10^th^edition (ICD-10) [19]. The DAD includes administrative, clinical, and demographic information on hospital discharges from all provinces and territories, except Quebec. NACRS contains information on care provided in the emergency department (ED) and other ambulatory care settings but was only available for Ontario and Alberta during the study time frame [20]. NACRS was only used as part of a sensitivity analysis described further below. The CVSD was linked to the DAD at the individual transactional level for fiscal years 2004/2005 to 2014/2015, allowing for a three-year observational window leading up to deaths which occurred on or after January 1, 2008. The observational window was required to determine the presence of relevant ICD10 codes for life-threatening conditions in the three years before death as well as the care provided in the last 90 days of life for those who died early in 2008. The linkage was enabled by Statistics Canada through use of a unique identifying number created in the Social Data Linkage Environment (SDLE), which is a collection of identifiers from different datasets. Individuals in the CVSD were linked to the SDLE, which in turn was linked to transactions in the DAD and NACRS. Linkage was done using date of birth, postal code, sex, and health insurance number. Decedents for which a good quality link could not be established were excluded from the cohort.

Those residing or dying in Quebec were excluded due to lack of coverage of this province in the DAD. Due to small sample sizes and unique features of healthcare provision in northern Canada, decedents residing or dying in the Yukon, Northwest Territories, and Nunavut were also excluded. Infants under three months of age were removed to avoid censoring in calculation of rates of health care use leading up to death [21]. To identify decedents with an underlying life-threating condition, we first excluded those with a primary cause of death in the CVSD listed as external (ICD10: V01-Y36; e.g., motor vehicle accident, suicide) or Sudden Infant Death Syndrome (R95). Then, based on a list of ICD10 codes (Supplemental File A) for life-threatening conditions [22, 23] and recommendations for maximizing the population size of those with life-threatening conditions using administrative data [24], we excluded those without a relevant ICD10 code listed as the primary or contributing causes of death or in the diagnostic codes recorded for each hospital admission over the three years prior to death.

Those with missing or likely erroneous data such as a date of death occurring months before last hospital admission were removed. Another potential data error included those listed in the CVSD as dying in hospital, but without a DAD record within three days of death. Since deaths in the ED would be coded as a hospital death in the CVSD but are recorded in NACRS rather than DAD, we assumed that these deaths occurred in the ED. In Ontario and Alberta, the only provinces with NACRS during the study timeframe [20], we excluded cases with no record in DAD or NACRS, within 3 days of the CVSD-reported hospital death. A sensitivity analysis (available on request) indicated minimal difference in findings with and without the excluded cases. As well, demographic characteristics were similar in the excluded and included cases except for geographic region, which may reflect differences in reporting across provinces.

### Measures

#### Outcomes

Indicators of high intensity EOL care were chosen based on previous studies of the intensity of EOL care in adults [25–27] and children [1] and the availability of data in the DAD. Indicators, created using DAD admission and discharge dates or intervention codes, included: 1) ≥ 1 ICU stay in the 30 and 14 days before death; 2) ≥ 2 hospital stays in the 30 and 14 days before death; 3) death in ICU; 4) use of mechanical ventilation in the 14 days before death; 5) in-hospital CPR in the 14 days before death; and 6) number of days in hospital over the last 30 and 90 days of life.

#### Demographic predictors

Sex and age were taken directly from the CVSD file. Postal Code Conversion File Plus was used to determine income quintiles according to residing neighbourhood at the time of death; rurality, with centres having a population < 10,000 classified as rural [28]; and distance from tertiary pediatric hospital using longitude and latitude data. For the latter, distance from the closest tertiary pediatric hospital was categorized into < 50 km, 50–199 km, 200–400 km, and > 400 km to represent increasingly more complex trips (i.e., an easy day trip both ways, a more substantial day trip both ways, a trip likely involving a stay overnight, and an overnight trip possibly involving a plane ride) [29].

#### Diagnostic predictors

Predictor variables were created for diagnostic categories consistent with a life-threating condition: neurology, haematology, oncology, metabolic, respiratory, circulatory, gastrointestinal, genitourinary, perinatal, congenital, and other (e.g., systemic lupus) (See Supplemental File A) [22, 23]. Decedents had to be assigned to at least one category but could be assigned to multiple categories. Consistent with previous research [16, 17] we also created a “diagnostic complexity” variable indicating whether the child had diagnoses in 1, 2, or 3 + categories.

#### Control variable

Region of residence was used as a control variable rather than a predictor to reduce the differences in data quality by province (e.g., higher frequencies of hospital deaths based on the CVSD than could be verified using the DAD). However, region of residence was reported in the demographic information with some provinces combined to avoid small cell sizes: Atlantic (Newfoundland and Labrador; Prince Edward Island, Nova Scotia, and New Brunswick), Ontario, Prairies (Manitoba and Saskatchewan), Alberta, and British Columbia.

### Statistical analysis

SAS (version 9.4) was used for all analyses. Descriptive statistics were produced to summarize demographic characteristics and prevalence of high intensity EOL care indicators. We used logistic regression to model the association between the predictor variables and each indicator of high intensity EOL care except number of hospital days where, negative binomial regression was used. Model predictors, selected a priori*,*included age, sex, income quintile, rurality, distance from tertiary pediatric centre, diagnosis, and diagnostic complexity. As full fit was desired, we left variables in each model regardless of *p*-value [30]. Model diagnostics were undertaken prior to selecting the final models for each outcome. We tested for multicollinearity using the threshold of VIF < 5. All logistic regression models had a significant Likelihood Ratio (Chi Square) test. To determine the appropriateness of using Negative Binomial regression vs. Poisson regression, we assessed the dispersion statistic. Region of residence was controlled for in all models. All statistical tests were two-sided; a value of *p* < 0.05 was used to establish statistical significance.

## Results

**Fig. 1:**
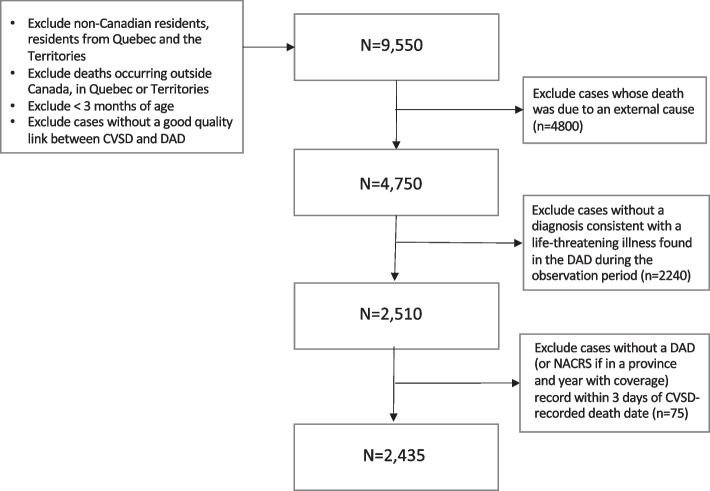
Flow chart of cohort selection

A total of 2435 children died from a life-threatening condition between January 1, 2008 and December 31, 2014 (Fig. 1). The three most common diagnostic categories were neurology (50.1%), oncology (38.0%), and congenital (35.9%) with 50.9% having diagnoses in ≥ 3 categories (Table 1). The prevalence of each high intensity EOL care indicator is provided in Table 2. Of note, 42.5% (*n* = 1035) children had at least one ICU stay in the last 30 days of life and 36.1% (*n* = 880) died there.

**Table 1:** Demographic characteristics of children who died from a life-threatening condition between January 1, 2008 and December 31, 2014 (*n* = 2435)

**Patient Characteristic**	**N(%)**
**Sex**	
Male	1295 (53.3)
Female	1135 (46.7)
**Age**	
3 months—1 year	380 (15.6)
1—4 years	600 (24.6)
5—9 years	420 (17.2)
10—14 years	410 (16.8)
15—19 years	625 (25.7)
**Diagnostic Categories**	
Neurology	1220 (50.1)
Haematology	720 (29.6)
Oncology	925 (38.0)
Metabolic	640 (26.3)
Respiratory	620 (25.5)
Circulatory	450 (18.5)
Gastrointestinal	295 (12.1)
Genitourinary	390 (16.0)
Perinatal	385 (15.8)
Congenital	875 (35.9)
Other	315 (12.9)
**Diagnostic Complexity**	
1 only	480 (19.7)
2 only	710 (29.2)
3 or more	1240 (50.9)
**Income Quintile**	
1 (lowest)	510 (21.2)
2	495 (20.6)
3	425 (17.7)
4	560 (23.3)
5 (highest)	415 (17.3)
**Region of Residence**	
Ontario	1205 (49.5)
Atlantic	200 (8.2)
Prairies	300 (12.3)
Alberta	425 (17.5)
British Columbia	305 (12.5)
**Rurality**	
Rural	500 (20.7)
Urban	1915 (79.3)
**Distance from Pediatric Hospital**	
< 50 km	1270 (52.6)
50–199 km	710 (29.4)
200–400 km	320 (13.3)
> 400 km	115 (4.8)

**Table 2:** Prevalence of indicators of high intensity end-of-life care (*n* = 2435)

**Indicator**	**N (%)**
ICU^a^ stay in last 30 days of life	1035 (42.5)
ICU^a^ stay in the last 14 days of life	975 (40.4)
Two or more hospital stays in the last 30 days of life	480 (19.7)
Two or more hospital stays in the last 14 days of life	255 (10.7)
Death in the ICU^a^	880 (36.1)
Use of mechanical ventilation in last 14 days of life	800 (32.9)
In-hospital cardiopulmonary resuscitation in the last 14 days of life	235 (9.7)
Median (IQR^a^) number of hospital days in the last 30 days of life	4 days (20 days)
Median (IQR^a^) number of hospital days in the last 90 days of life	10 days (34 days)

^a^ *ICU* Intensive Care Unit, *QR* interquartile range

The odds significantly associated with each indicator of high intensity EOL care according to the demographic and diagnostic predictors are found in Table 3. Among demographic predictors, age was most consistently associated with indicators. Decedents aged 3 months to less than 1 year had higher odds of ICU stays (30 days: OR = 2.95, 95% CI = 2.11–4.11; 14 days: OR = 2.69, 95% CI = 1.93–3.75), ICU death (OR = 2.33, 95% CI = 1.66–3.27), mechanical ventilation (OR = 2.49, 95% CI = 1.78–3.48) within 2 weeks of death and more days in hospital (30 days: RR = 1.99, 95% CI = 1.58–2.50; 90 days: RR = 2.91, 95% CI = 2.33–3.63) compared to the oldest cohort members. By contrast, while the effects were smaller, those aged 5 to 9 years had lower odds of the above-mentioned indicators, as well as lower odds of multiple hospital stays 30 (OR = 0.71, 95% CI = 0.51–0.97) and 14 days before death (OR = 0.57, 95% CI = 0.37–0.89), compared to the oldest group. Those aged 10 to14 years also had lower odds of multiple hospital stays in the 30 (OR = 0.59, 95% CI = 0.42–0.83) and 14 (OR = 0.50, 95% CI = 0.32–0.79) days before death.

**Table 3:** Odds or rate ratios* and 95% confidence intervals associated with indicators of high-intensity care (Only ratios significant at *p* < 0.05 presented) (*n* = 2435)

**Predictor**	**Category**	**ICU stay (30 days)**	**ICU stay(14 days)**	** > 1 hospital stay (30 days)**	** > 1 hospital stay (14 days)**	**ICU death**	**Mechanical Ventilation (14 days)**	**CPR (14 days)**	Hospital days (30 days)*	Hospital days (90 days)*
**Age** ref = 15–19 years	3 months—< 1 year	2.95 (2.11–4.11)	2.69 (1.93–3.75)	ns	ns	2.33 (1.66–3.27)	2.49 (1.78–3.48)	ns	1.99 (1.58–2.50)	2.91 (2.33–3.63)
	1–4 years	ns	ns	ns	ns	ns	ns	ns	ns	
	5–9 years	0.68 (0.51–0.91)	0.65 (0.49–0.88)	0.71 (0.51–0.97)	0.57 (0.37–0.89)	0.72 (0.53–0.99)	0.61 (0.44–0.83)	ns	0.71 (0.59–0.86)	0.75 (0.63–0.91)
	10–14 years	ns	ns	0.59 (0.42–0.83)	0.50 (0.32–0.79)	ns	ns	ns	ns	ns
**Income** ref = 5th quintile (highest)	1st quintile (lowest)	ns	ns	ns	ns	ns	ns	ns	1.25 (1.02–1.52)	1.33 (1.10–1.61)
	2nd quintile	ns	ns	ns	ns	ns	ns	ns	ns	ns
	3rd quintile	ns	ns	ns	ns	ns	ns	0.55 (0.32–0.93)	ns	ns
	4th quintile	ns	ns	ns	ns	ns	ns	ns	ns	ns
**Rurality**	Rural	ns	ns	ns	ns	ns	ns	ns	ns	ns
**Sex**	Female	ns	ns	ns	ns	ns	ns	ns	ns	ns
**Distance from pediatric hospital** ref = < 50 km	50–99 km	0.75 (0.60–0.94)	ns	ns	ns	ns	ns	ns	ns	ns
	200–400 km	ns	ns	1.62 (1.16–2.27)	1.59 (1.03–2.44)	ns	ns	ns	ns	ns
	> 400 km	ns	ns	2.09 (1.33–3.33)	1.94 (1.08–3.46)	ns	ns	2.11 (1.15–3.90)	ns	ns
Diagnosis**	Neurology	ns	ns	0.77 (0.59–1.0)	ns	ns	ns	0.67 (0.47–0.96)	ns	0.84 (0.73–0.98)
	Haematology	ns	ns	ns	0.59 (0.39–0.82)	ns	ns	ns	1.33 (1.10–1.61)	1.45 (1.21–1.73)
	Oncology	0.47 (0.36–0.62)	0.41 (0.31–0.55)	1.37 (1.01–1.88)	ns	0.37 (0.28–0.50)	0.36 (0.27–0.49)	0.36 (0.22–0.59)	1.38 (1.16–1.65)	1.61 (1.36–1.91)
	Metabolic	0.75 (0.59–0.94)	0.69 (0.54–0.87)	ns	ns	0.69 (0.54–0.89)	0.69 (0.54–0.82)	0.59 (0.40–0.88)	ns	ns
	Respiratory	2.10 (1.67–2.65)	2.03 (1.61–2.56)	ns	1.39 (1.00–1.92)	2.08 (1.64–2.64)	2.04 (1.60–2.59)	1.45 (1.02–2.07)	1.56 (1.33–1.82)	1.53 (1.32–1.78)
	Circulatory	1.85 (1.43–2.40)	1.80 (1.39–2.33)	ns	ns	1.98 (1.53–2.57)	1.75 (1.35–2.27)	2.95 (2.07–4.21)	ns	ns
	Gastrointestinal	1.66 (1.24–2.24)	1.76 (1.31–2.37)	1.38 (1.01–1.89)	ns	1.98 (1.47–2.68)	1.66 (1.23–2.24)	ns	1.25 (1.03–1.52)	ns
	Genitourinary	3.42 (2.60–4.51)	3.41 (2.60–4.49)	ns	ns	4.08 (3.09–5.39)	3.42 (2.60–4.51)	1.91 (1.30–2.82)	1.48 (1.24–1.77)	1.47 (1.24–1.75)
	Perinatal	0.67 (0.50–0.91)	0.66 (0.49–0.89)	0.68 (0.47–0.96)	ns	0.66 (0.49–0.89)	ns	ns	ns	ns
	Congenital	ns	ns	ns	ns	ns	0.66 (0.51–0.86)	ns	ns	ns
	Other	ns	0.51 (0.41–0.64)	ns	ns	0.64 (0.47–0.88)	0.70 (0.52–0.96)	0.49 (0.29–0.84)	ns	1.27 (1.06–1.53)
**Diagnostic Complexity** (ref = 1 diagnosis)	2 diagnoses	ns	ns	ns	ns	ns	ns	ns	1.51 (1.24–1.84)	1.60 (1.33–1.94)
	3 or more diagnoses	ns	ns	2.08 (1.29–3.35)	1.87 (1.01–3.44)	ns	ns	ns	1.65 (1.25–2.19)	1.86 (1.42–2.44)

^*^Rate ratios expressed for number of hospital days in the last 30 and 90 days of life; Odds ratios expressed for all other indicators

^**^Diagnosis was represented by a set of indicators (0/1) for each possible diagnosis

Living further away from a tertiary pediatric hospital was associated with higher odds of multiple hospitalizations 30 and 14 days before death. Those furthest away (> 400 km) compared to closest (< 50 km) also had higher odds of in-hospital CPR in the 14 days before death (OR = 2.11, 95% CI = 1.15–3.90). Those 50–99 km from the pediatric centre had lower odds of ICU admission 30 days before death (OR = 0.75, 95% CI = 0.60–0.94); no effect was found for the 14-day version of the indicator.

Higher rates of hospital days in the 30 and 90 days before death were found in the lowest income level compared to the highest (30 days: RR = 1.25, 95% CI = 1.02–1.52; 90 days: RR = 1.33, 95% CI = 1.10–1.61). Sex and rurality were not associated with any high intensity EOL care indicator.

Each diagnosis was associated with at least one high intensity indicator, but strength and direction varied. Respiratory, circulatory, gastrointestinal, and genitourinary diagnoses were associated with higher odds of at least five different high intensity indicators including ICU stays in the last 30 and 14 days of life, ICU death, use of mechanical ventilation, and number of hospital days in the last 30 and 90 days of life. The largest odds ratios were observed for genitourinary diagnoses. By contrast, oncology and metabolic conditions were associated with lower odds of ICU stays, death in ICU, use of mechanical ventilation, and use of CPR in hospital, with the largest effects found for oncology. However, those with oncology conditions had higher number of hospital days in the last 30 and 90 days of life. Those with three or more diagnoses had higher odds of multiple hospitalizations within both 30 (OR = 2.08, 95% CI = 1.29–3.35) and 14 days (OR = 1.87, 95% CI = 1.01–3.44) of death and spent more time in hospital (30 days: RR = 1.65, 95% CI = 1.25–2.19; 90 days: RR = 1.86, 95% CI = 1.42–2.44) than those with a single diagnosis.

## Discussion

Our findings highlight the large number of children who died from non-cancer diagnoses in Canada and the high prevalence of indicators of high intensity EOL care. High intensity care was associated with several predictors including younger age (less than 1), longer distance from a tertiary hospital (> 400 km), lower income, diagnostic category (e.g., respiratory and genitourinary), and higher diagnostic complexity.

The most common diagnostic category in our study was neurology (51.1%). Neurological conditions were also identified as the most common category of life-threatening conditions in research conducted across 40 pediatric hospitals in the United States (46%) [16] and in California (46%) [17] for children aged 1 to 18 years. Meanwhile, congenital conditions were identified as the most common diagnosis in a study across the United Kingdom focused on children from birth to 19 years living with life-threatening conditions; highlighting the differences in prevalence of diagnoses based on the child’s age [31]. While cancer was among the top three most common diagnostic categories across all four studies, the prevalence of non-cancer diagnoses highlights the importance of examining all children with life-threatening conditions. This larger population includes many children with multiple diagnoses: with 50.9% having diagnoses in three or more categories in our study compared with 34% across the United States [16] and 42% in California [17]. The higher proportion of multiple diagnoses in Canadian children may reflect differences in how diagnoses are recorded and how many fields were searched for relevant diagnoses among the studies [24].

Children with cancer in our sample had reduced odds of experiencing all high intensity indicators except hospital stays where they had increased odds of having more than one hospital stay in the last 30 days of life and spent more days in hospital in the last 30 and 90 days of life. Previous research in Ontario, Canada focused only on children with cancer found a similar proportion of children with more than one hospital stay in the last 30 days of life (17.6% vs 19.7%), but a much lower proportion of children who stayed in ICU during the last 30 days of life (21.7% vs 42.5%) which may reflect some provincial differences in the way care is provided or the resources available [1]. The vast majority of research to date has focused on children with cancer, yet our study demonstrates that high intensity EOL care is more prevalent in children with non-cancer diagnoses. Those with genitourinary conditions had the highest odds of most high-intensity indicators. These conditions included both chronic and acute kidney disease which may have necessitated admission to hospital or ICU for dialysis, particularly in the face of comorbid conditions. It is critically important to further study children with the full breadth of life-threatening conditions to better understand the impact of differences in disease trajectory particularly with the rising prevalence of rare diseases and medical complexity in children [31, 32] and challenges with prognostication [33] which may result in different opportunities to enhance the care provided across disease groups.

Several studies have noted the high reliance on medical technology, as well as high use and cost of health care throughout illness in children with complex chronic conditions [17, 32, 34–36], many of which are also life-threatening. In the last 30 days of life, 33.6% (2911/8654) of children with a complex chronic condition, age 1- 21 years who died in California between 2000 and 2013 study were admitted to the ICU [17] while 42.5% of our sample had an ICU stay. The higher proportion of ICU stays in our study is likely due to inclusion of children < 1 year of age, who were nearly 3 times more likely to have an ICU stay than adolescents. The proportion of children in our study who received CPR (9.7%) was similar to that reported in the California study (11%; 969/8654). Both studies only captured CPR use in the hospital setting thus are likely underestimates given that CPR may have been provided at home or in the ED. However, if the child was in hospital, there should have been more time for discussion of its use, while providing CPR in other locations may have been due to an acute change in status and thus in line with goals of care.

Some research has found an association between low income and high intensity care at the EOL both in countries with primarily private [4, 17] and socialized health care systems [3, 21]. However, in our sample, those in the lowest income quintile did not have increased odds of experiencing any high intensity EOL care indicators except higher rates of hospital days over the last 30 and 90 days of life. Similarly, research focused on children living with complex chronic conditions found those with lower scores on the Child Opportunity Index (a multidimensional measure of 29 indicators related to education, health and environment, and social and economic opportunity) spent more time in hospital [37]. Increased resources both at home and in the hospital specifically designed for children from low-income areas may enable them to spend more time at home [37]. Further examination of home care use in this population may also uncover additional opportunities for improvement.

While 50% of the cohort lived within 50 km of a pediatric hospital, nearly 20% lived 200 km or further, meaning a visit to the pediatric hospital likely required an overnight stay. These children were more likely to have multiple admissions in the last 15 and 30 days of life than those who lived close to the hospital. It is possible children were admitted to hospital to facilitate conduct of various tests and meeting multiple specialists over a few days, while children living closer could more easily make several outpatient visits to receive the same care. As well, a lack of comprehensive home care for children living further away from the tertiary centre may have necessitated more frequent hospital admissions to provide care. Data on home care and outpatient visits are not available nationally [20] but should be further examined provincially, where possible, to understand interactions with the health system across care sectors.

While spending less time in hospital is typically associated with higher quality care [16, 26], it is possible that being in hospital offered greater opportunities for discussion and planning among the child, family, and clinicians which reduced the odds of experiencing other indicators of high intensity care at the EOL. Children with cancer and those with greater diagnostic complexity spent more time in hospital and those with the greatest complexity (3 or more diagnoses) had higher odds of more than one hospital stay in the last 14 and 30 days of life yet did not have higher odds of other high intensity interventions. Those with cancer had lower odds of high intensity care. Children with cancer may have a more predictable trajectory towards death particularly if treatments with curative intent have been stopped, potentially allowing for discussion about also forgoing high intensity interventions. For children with other life-threatening conditions with less predictable trajectories, a trial of high intensity interventions may be appropriate to help a child through an unexpected event (e.g., pneumonia) with the possibility of resumption of baseline functioning [33]. Unfortunately, administrative data do not provide information about any discussions on location or intensity of care and whether these indicators aligned with families’ goals of care. Similarly, identifying the involvement of specialized pediatric palliative care services, which may increase the likelihood of having these discussions and may differ by disease group, is not currently available in a reliable and valid way in administrative data [38]. Additional research is needed to examine these relationships and uncover further explanations for study findings.

## Limitations

While our goal was to create a national picture of EOL care for children with life-threatening conditions, we were limited by a lack of available data from Quebec, Yukon, Northwest Territories, and Nunavut. We also had to limit our examination to care provided in acute care settings, as databases that capture ambulatory care setting, including the ED, were only available in Ontario and Alberta at the time of analysis. However, this study provides important baseline data for future work particularly to support more detailed analyses in each province with access to a wide number of variables such as ED care, home care, physician visits, and palliative care service provision, particularly in the home, which may have a significant impact on the opportunity to receive care outside of a hospital setting [39] and which may help to explain difference seen by age, disease groups, and income. Finally, our results are subject to biases associated with multiple comparisons and use of a decedent cohort design to evaluate patterns in EOL care [40].

## Conclusion

Given the increasing prevalence of children living with life-threatening conditions [31] it is important to examine EOL care provided across the population, not just for those with cancer. Children with non-cancer diagnoses experienced higher odds of high intensity EOL care than those with cancer. While high intensity EOL care may be appropriate in some situations [26], it should not occur as the default particularly when the high intensity interventions are likely to increase the child’s suffering with little chance of benefit. Thoughtful care aligned with families’ goals balanced with the medical reality of child’s condition can help optimize resource utilization to best meet families’ needs. Exploration of sources of support such as home care or palliative care services in both home and hospital, as well as the type and outcomes of discussions that occur near the EOL, are needed to better understand experiences, and identify opportunities for improvement particularly in the non-cancer population.

## Supplementary Information

**Additional file 1:** Supplemental File A – ICD-10 Codes Signifying a Life-Threatening Condition [17, 18]. 

## Data Availability

The study protocol and programing code is available from the corresponding author (KW) upon request. Access to The Canadian Vital Statistic Database and Discharge Abstracts Database is available through Statistics Canada (https://www.statcan.gc.ca/en/about/relevant/vscc/access).

## Abbreviations

ICU: Intensive care unit
CPR: Cardiopulmonary resuscitation
EOL: End-of-life
CVSD: Canadian Vital Statistics Database
DAD: Discharge Abstracts Database
ICD-10: International Classification of Diseases, 10^th^ edition
SDLE: Social Data Linkage Environment
ED: Emergency department
NACRS: National Ambulatory Care Reporting System
OR: Odds Ratio
CI: Confidence Interval
RR: Rate Ratio
