# Supplementary material for: Intensity of end-of-life care among children with life-threatening conditions: a national population-based observational study
Source: BMC Pediatr. 2023 Jul 24;23:375. doi: 10.1186/s12887-023-04186-9 (PMC10364373; doi:10.1186/s12887-023-04186-9)
Supplement: Supplementary file 1 — Additional file 1: Supplemental File A – ICD-10 Codes Signifying a Life-Threatening Condition [17, 18]. [file 12887_2023_4186_MOESM1_ESM.docx]

| **Neurology** | | **Haematology** | **Oncology** | **Metabolic** | **Respiratory** | **Circulatory** | **GI*** | **GU*** | **Peri-natal** | **Congenital** | | **Other** |
| --- | --- | --- | --- | --- | --- | --- | --- | --- | --- | --- | --- | --- |
| A17 | G60.0 | B20-B24 | C00-C97 | E31.0 | E84 | I21 | K55.0 | N17 | P5 | Q00-Q07 | Q92.4 | H11.1 |
| A81.0 | G60.1 | D56.1 | D33 | E34.8 | G47.3 | I27 | K55.9 | N18 | P7.0 | Q20 | Q92.7 | H49.8 |
| A81.1 | G70.2 | D61.0 | D43 | E70.2-70.4 | J84.1 | I42-I45 | K72 | N19 | P7.2 | Q21.2-Q24 | Q92.8 | H35.5 |
| F71-F73 | G70.9 | D61.9 | D44.4 | E71 | J96 | I50.9 | K74 | N25.8 | P10.0 | Q25.1-Q26 | Q93 | M31.3 |
| F84.2 | G71.0-G71.3 | D70 | D48 | E72 | J98.4 | I51.5 | K76.5 |  | P10.1 | Q28.2 | Q95.0 | M32.1 |
| G10 | G71.8 | D76.1 |  | E74-E78 |  | I51.7 | K86.8 |  | P10.4 | Q28.3 | Q95.2 | M33.9 |
| G11.1-G11.4 | G71.9 | D81 |  | E79.1 |  | I51.8 |  |  | P11.2 | Q28.9 | Q96.9 | M34.0 |
| G11.8 | G72 | D82.1 |  | E83.0 |  | I61.3 |  |  | P11.5 | Q30-34 |  | M35.9 |
| G11.9 | G80 | D83 |  | E88 |  | I63.1 |  |  | P21.0 | Q39.0-39.4 |  | M89.5 |
| G12 | G81.9 | D89.1 |  |  |  | I63.2 |  |  | P21.9 | Q39.6 |  | T86.0 |
| G20 | G82.3-G82.5 |  |  |  |  | 163.3 |  |  | P25 | Q41-Q45 |  | T86.2 |
| G21.0 | G82.9 |  |  |  |  | I63.5 |  |  | P28.0 | Q60-Q64 |  | Z51.5† |
| G21.1 | G83.5 |  |  |  |  | I81 |  |  | P28.5 | Q74.3 |  |  |
| G21.8 | G83.9 |  |  |  |  |  |  |  | P29.0 | Q75.0 |  |  |
| G23.0-G23.2 | G90.1 |  |  |  |  |  |  |  | P29.3 | Q76.1 |  |  |
| G23.8 | G90.9 |  |  |  |  |  |  |  | P35.0 | Q77 |  |  |
| G24.0 | G91.1 |  |  |  |  |  |  |  | P35.1 | Q78 |  |  |
| G24.8 | G93.1 |  |  |  |  |  |  |  | P35.8 | Q79.0-Q79.5 |  |  |
| G25.3-G25.5 | G93.4-G93.9 |  |  |  |  |  |  |  | P37.1 | Q79.9 |  |  |
| G25.8 | G94 |  |  |  |  |  |  |  | P52.4 | Q80.4 |  |  |
| G25.9 | G95.1 |  |  |  |  |  |  |  | P52.5 | Q81 |  |  |
| G31.0 | G95.8 |  |  |  |  |  |  |  | P52.8 | Q82.1 |  |  |
| G31.8 |  |  |  |  |  |  |  |  | P52.9 | Q82.4 |  |  |
| G31.9 |  |  |  |  |  |  |  |  | P56.0 | Q85.1 |  |  |
| G32.8 |  |  |  |  |  |  |  |  | P57 | Q85.8 |  |  |
| G35 |  |  |  |  |  |  |  |  | P61.3 | Q86.0 |  |  |
| G37.1 |  |  |  |  |  |  |  |  | P61.4 | Q87.0-87.4 |  |  |
| G37.2 |  |  |  |  |  |  |  |  | P77 | Q87.8 |  |  |
| G37.8 |  |  |  |  |  |  |  |  | P83.2 | Q89.7 |  |  |
| G40.1-G40.5 |  |  |  |  |  |  |  |  | P91.2 | Q89.9 |  |  |
| G40.8 |  |  |  |  |  |  |  |  | P91.6 | Q91 |  |  |
| G40.9 |  |  |  |  |  |  |  |  | P96.0 | Q92.0-92.2 |  |  |

**Supplemental File A – ICD-10 Codes Signifying a Life-Threatening Condition^17,18^**

*GI = Gastrointestinal; GU = Genitourinary

†When assigning diagnostic complexity, the code for palliative care (Z51.1) was only used if it was the sole relevant diagnostic code for a child.

Table accepted for publication as supplementary material in: Widger, K., Brennenstuhl, S., Tanuseputro, P., Nelson, K., Rapoport, A., Seow. H., Siden, H., Vadeboncoeur, C., Gupta, S. (Accepted November 2022). Location of death among children with life-threatening conditions: A national population-based observational study using the Canadian Vital Statistics Database (2008 – 2014). *CMAJ Open.*
